# Publisher Correction: Inference of transcription factor binding from cell-free DNA enables tumor subtype prediction and early detection

**DOI:** 10.1038/s41467-020-15799-4

**Published:** 2020-04-20

**Authors:** Peter Ulz, Samantha Perakis, Qing Zhou, Tina Moser, Jelena Belic, Isaac Lazzeri, Albert Wölfler, Armin Zebisch, Armin Gerger, Gunda Pristauz, Edgar Petru, Brandon White, Charles E. S. Roberts, John St. John, Michael G. Schimek, Jochen B. Geigl, Thomas Bauernhofer, Heinz Sill, Christoph Bock, Ellen Heitzer, Michael R. Speicher

**Affiliations:** 1 Institute of Human Genetics, Diagnostic and Research Center for Molecular BioMedicine, Medical University of Graz, Graz, Austria; 2 Department of Internal Medicine, Division of Hematology, Medical University of Graz, Graz, Austria; 3 Department of Internal Medicine, Division of Oncology, Medical University of Graz, Graz, Austria; 4 Department of Obstetrics and Gynecology, Medical University of Graz, Graz, Austria; 5 Freenome, South San Francisco, CA USA; 6 Institute of Medical Informatics, Statistics and Documentation, Medical University of Graz, Graz, Austria; 7 CeMM Research Center for Molecular Medicine of the Austrian Academy of Sciences, Vienna, Austria; 8 Department of Laboratory Medicine, Medical University of Vienna, Vienna, Austria; 9 Max Planck Institute for Informatics, Saarland Informatics Campus, Saarbrücken, Germany; 10 BioTechMed-Graz, Graz, Austria; 11 Christian Doppler Laboratory for Liquid Biopsies for Early Detection of Cancer, Graz, Austria

**Keywords:** Oncology, Cancer, Tumour biomarkers

Correction to: *Nature Communications* 10.1038/s41467-019-12714-4, published online 11 October 2019.

The original version of this Article contained errors in Figure 1 and Figure 4. In Fig. 1b the graph of the lower right panel showing the DNAse hypersensitivity coverage was duplicated from the upper right panel.

In Fig. 4a the label of the second panel of the left figure was incorrectly labelled DLx2 rather than DLX2. In addition, the label of the third panel of the left figure of Fig. 4a was labelled EVx2 rather than EVX2.

These errors have now been corrected in both the PDF and HTML versions of the article.

