# Supplementary material for: Inference of transcription factor binding from cell-free DNA enables tumor subtype prediction and early detection
Source: Nat Commun. 2019 Oct 11;10:4666. doi: 10.1038/s41467-019-12714-4 (PMC6789008; doi:10.1038/s41467-019-12714-4)
Supplement: Supplementary file 1 — Supplementary Information [file 41467_2019_12714_MOESM1_ESM.pdf]

Supplementary Information

Inference of transcription factor binding from cell-free DNA enables tumor subtype prediction and early detection

Ulz et al.

Supplementary information

Supplementary Figure 1....................3
Supplementary Figure 2....................4
Supplementary Figure 3....................5
Supplementary Figure 4....................6
Supplementary Figure 5....................7
Supplementary Figure 6....................8
Supplementary Figure 7....................9
Supplementary Figure 8....................10
Supplementary Figure 9....................11
Supplementary Table 1....................12
Supplementary Table 2....................13
Supplementary Table 3....................14

Supplementary Figure 1

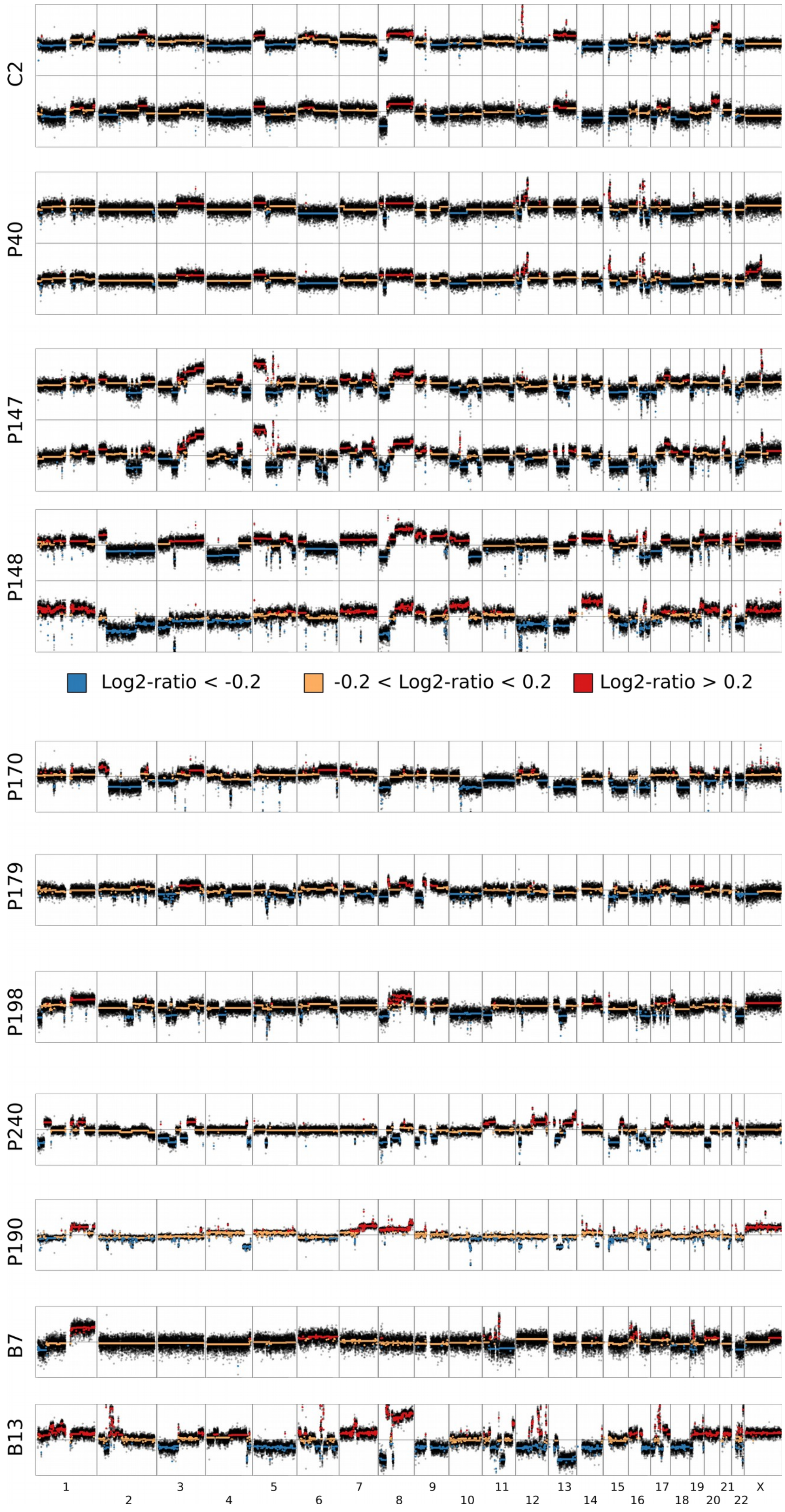

Somatic copy number alterations (SCNAs) in plasma samples from patients with cancer

SCNAs identified after whole-genome plasma sequencing of plasma samples derived from one patient with colon adenocarcinoma (C2); eight patients with prostate cancer (P40, P147, P148, P170, P179, P198, P240, P190); and two patients with breast cancer (B7, B13).

Supplementary Figure 2

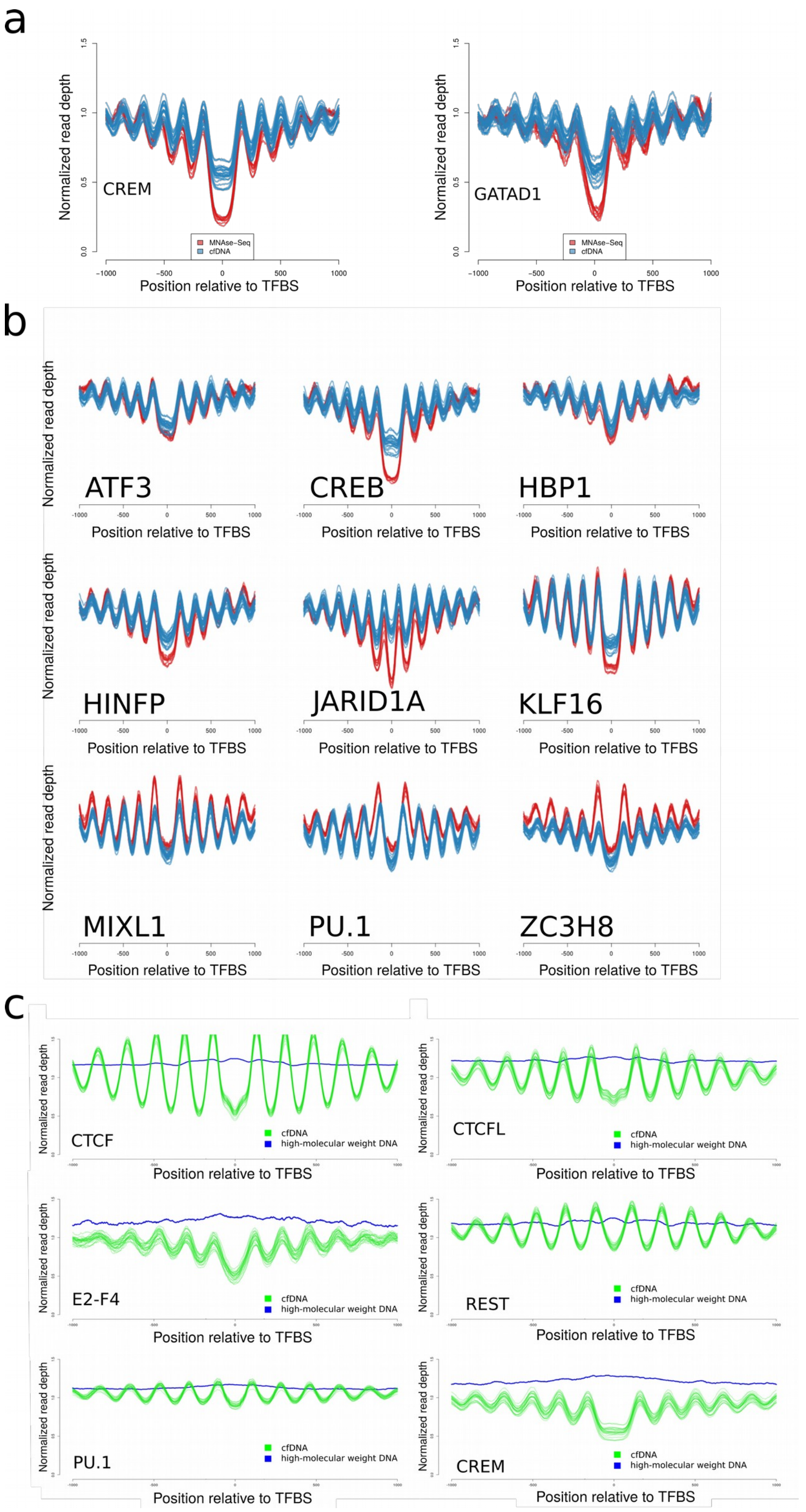

a) TFBS-nucleosome coverage profiles for two representative TFs, CREM and GATAD1, established from 24 cfDNA samples from healthy controls, each shown with an individual blue line. The MNase-seq coverage patterns from the lymphoblastoid cell line GM12878 obtained from ENCODE are illustrated in red. **b)** Additional comparisons between coverage profiles of cfDNA and MNase-seq around TFBSs. **c)** TFBS analyses with high-molecular weight DNA, which is not mono-nucleosomal DNA, yields a uniform, non-oscillating pattern (blue) in contrast to plasma DNA (green).

Supplementary Figure 3

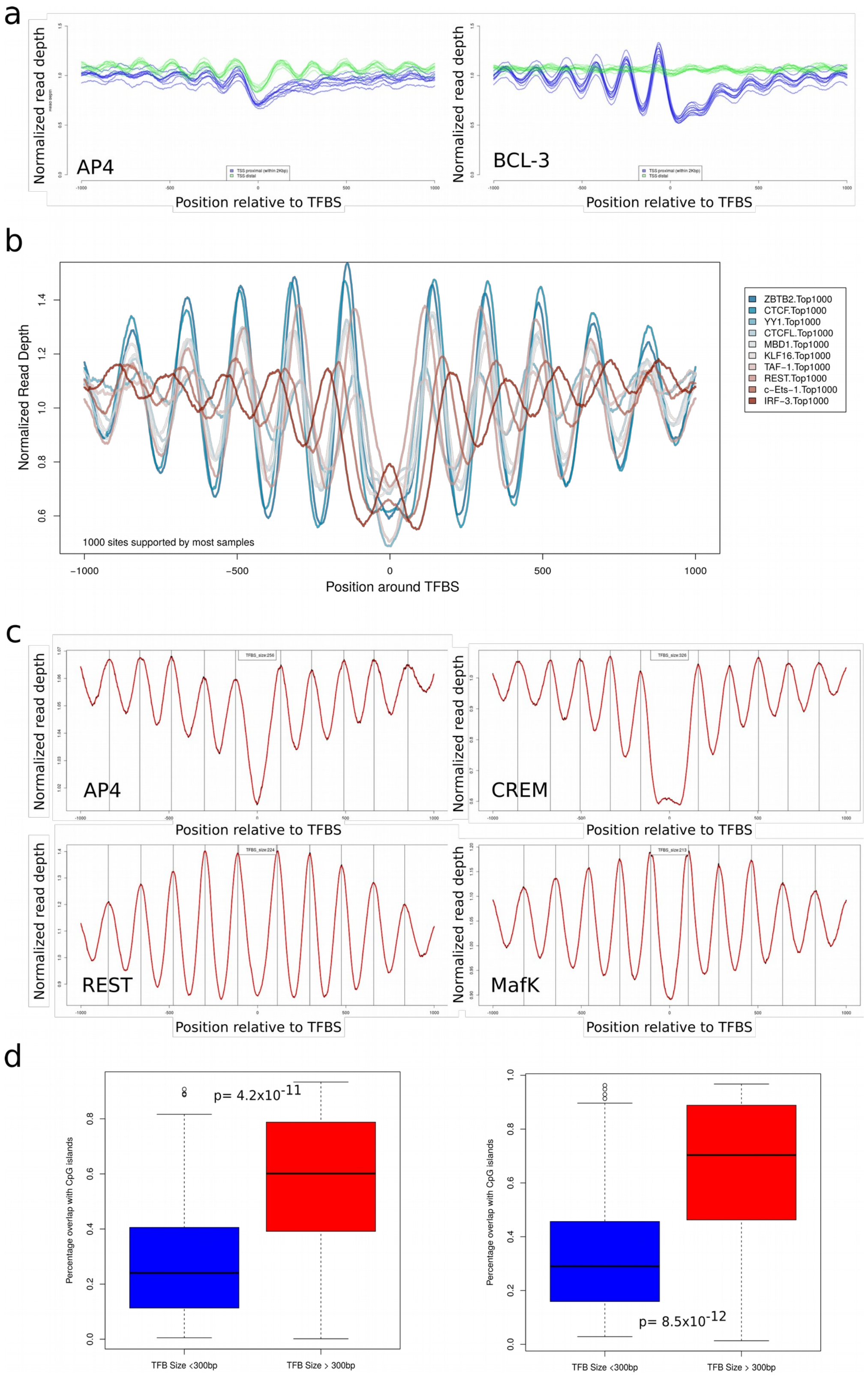

a) Coverage profiles for TFs AP-4 and BCL-3 after calculations conducted separately for TFBS within and outside of TSSs. Profiles within TSSs are oriented ina way that the actual transcription start at 0 in the positive direction. **b)** Exemplary TF-nucleosome profiles illustrating the variable nucleosome patterns of different TFs in cfDNA. **c)** Measurements of TFBS widths revealed substantial differences among various TFBSs. **d)** Boxplot illustrating the percentage of overlap for CpG islands (left panel) and TSSs (right panel).

Supplementary Figure 4

Normal distribution of z-scores

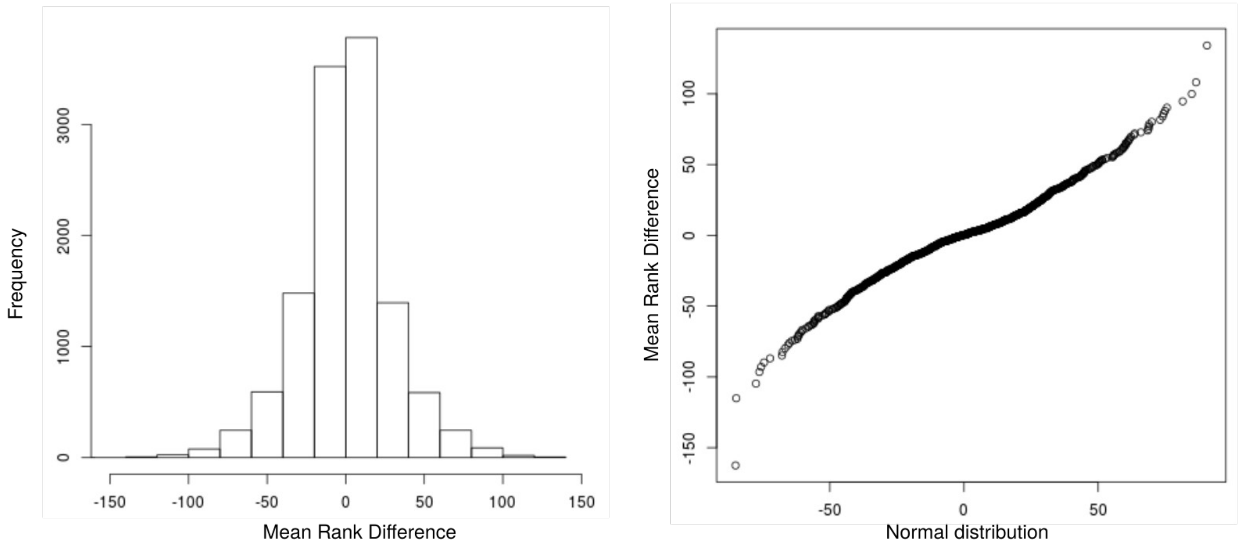

Rank differences of each healthy sample against the remaining healthy samples shows normal distribution as depicted by its distribution (left panel), as well as the QQ-plot (right panel).

Supplementary Figure 5

Overall z-scores in high-coverage tumor samples

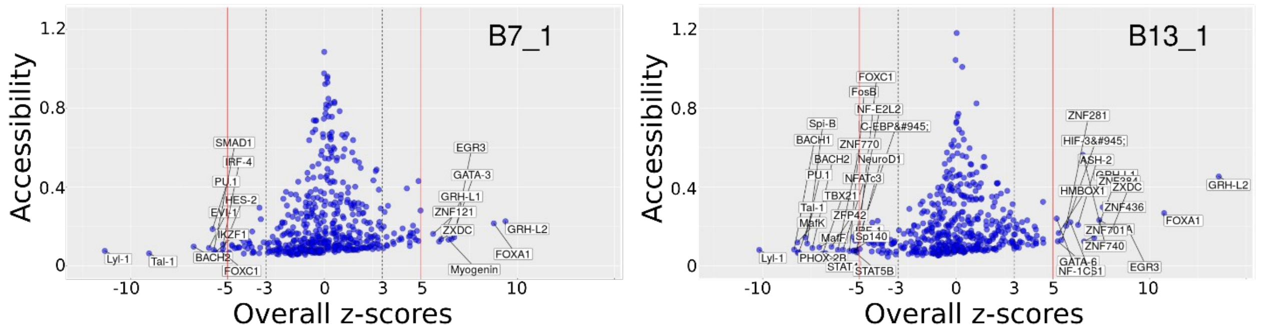

In breast cancer samples B7 and B13 we detected in agreement with the ATAC-seq data increased accessibility for GRHL2, FOXA1, and ZNF121.

Supplementary Figure 6

TFBS accessibility in serial analysis

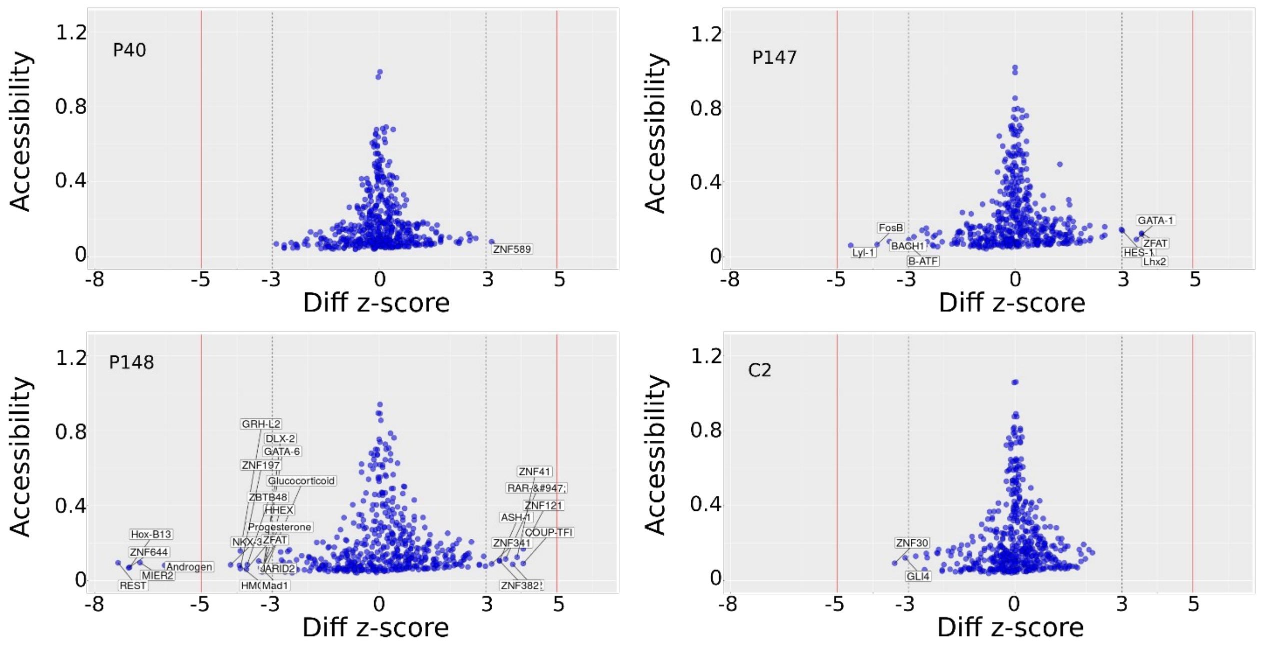

Pairwise comparison of two plasma samples from patients C2, P147, and P40.

Supplementary Figure 7

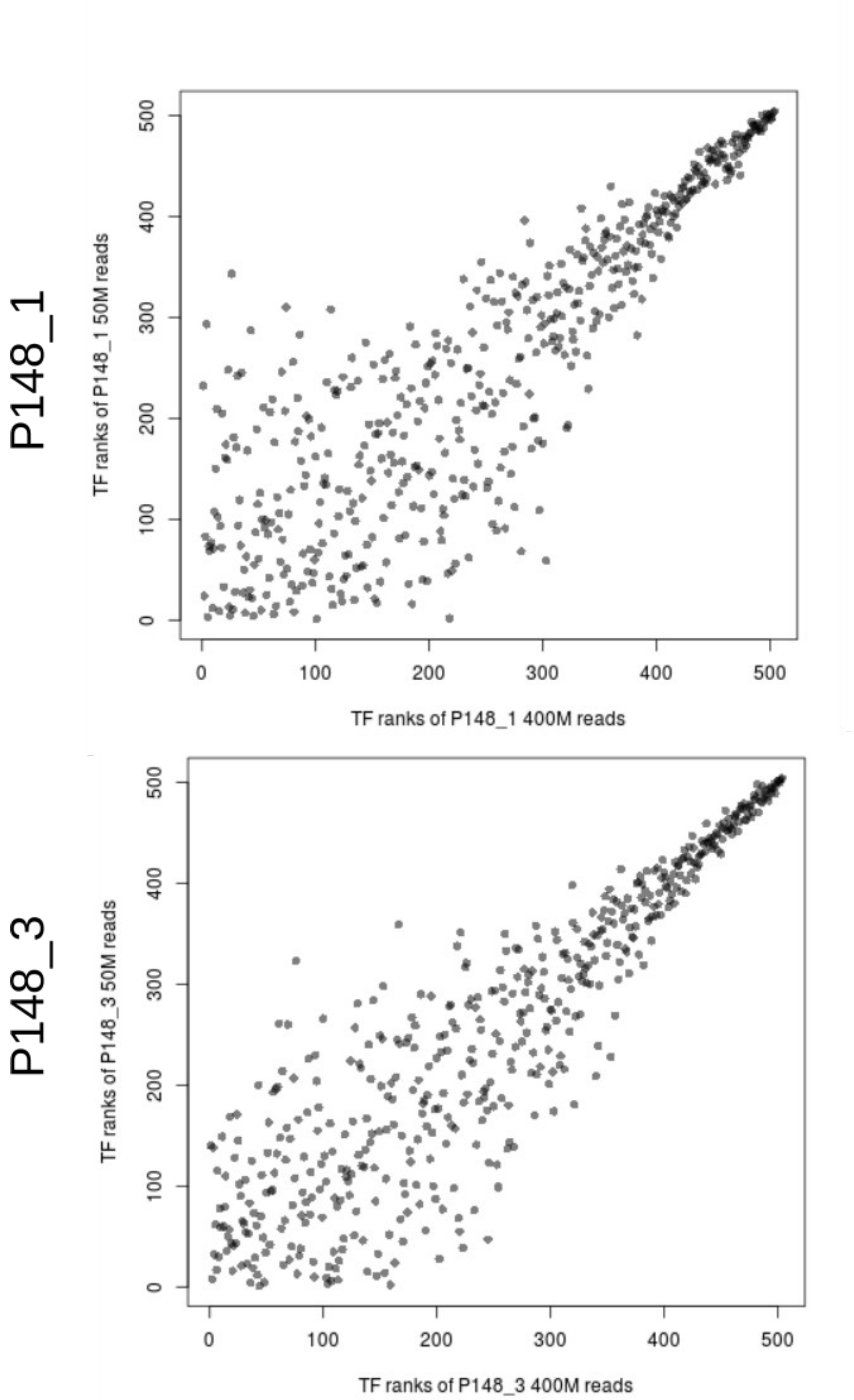

Down-sampling of plasma samples P148_1 and P148_3

Plasma samples P148_1 (819,607,690 reads) and P148_3 (768,763,081 reads) were down-sampled to ~50 million reads and analyzed for the 504 TFs used in our study. Lower ranking TFs appear to be more affected by noise.

Supplementary Figure 8

Determination of the tumor content with ichorCNA

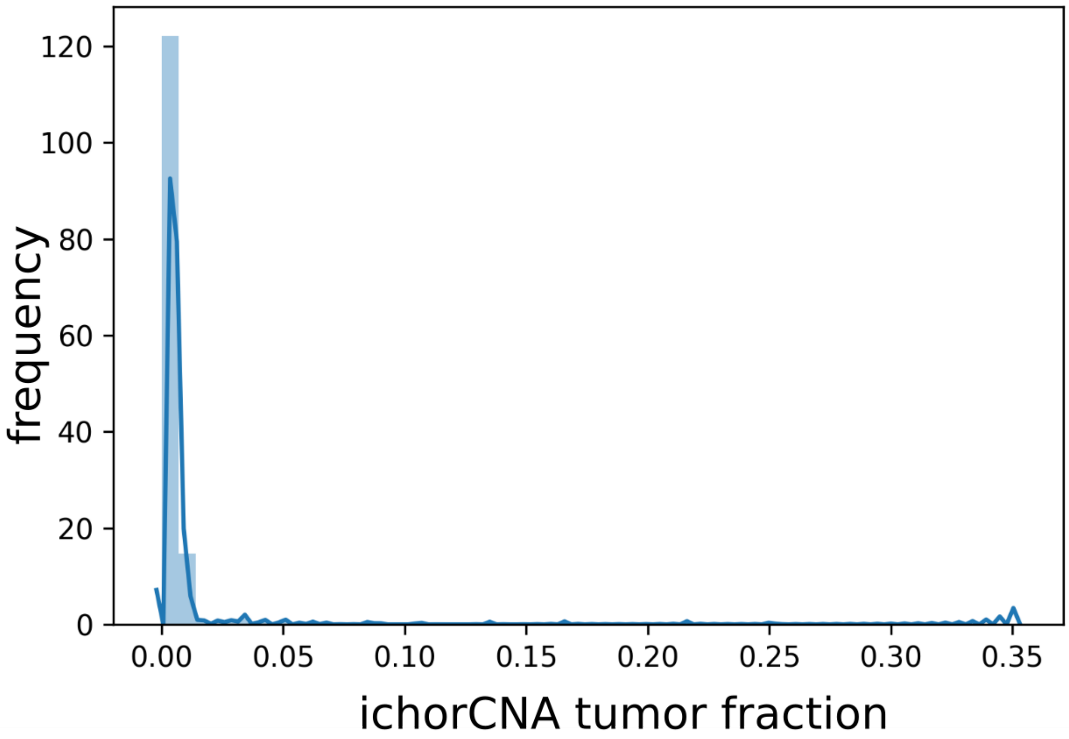

Distribution of tumor fractions as measured by ichorCNA in the Freenome colon cancer cohort. Most samples show a tumor fraction below the limit of detection specified by ichorCNA (3%).

Supplementary Figure 9

Logistic Regression on all samples from the colon cancer cohort, as well as samples with stage I and II combined

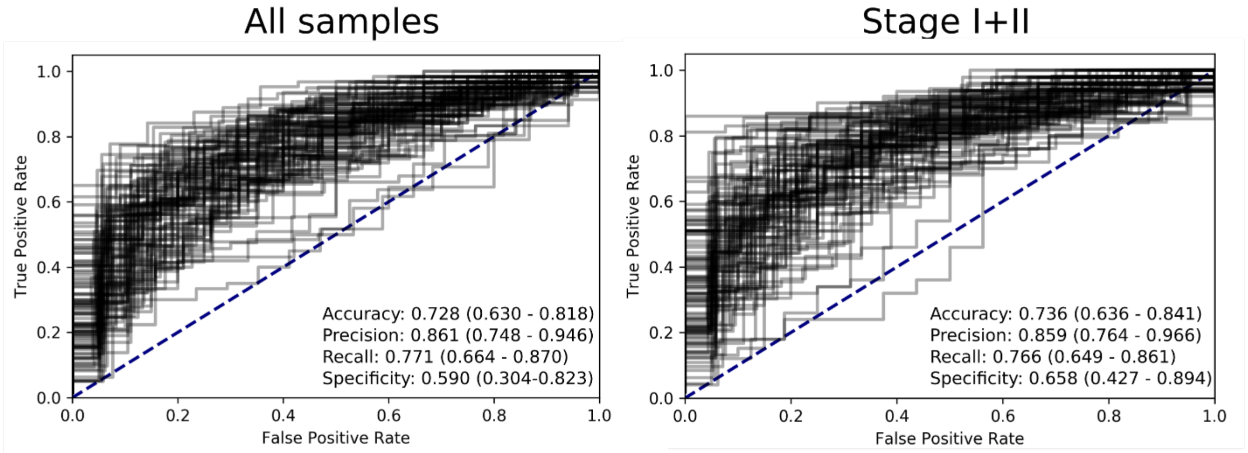

ROC-curves of logistic regression on 100 random training-test splits on accessibilities of 504 samples. The left panel shows all cancer samples, irrespective of stage, combined into a single class, while the right panel show samples at stages I and II. Additional performance metrics are available in Supplementary Table 3.

Prostate cancer patients

Patient ID	Sample ID	Age (years)	Tumor type	Metastatic sites	Castratation Resistance	PSA at diagnosis [ng/ml]	PSA [ng/ml]	NSE [ng/ml]	Treatment	Previous treatment	AR Amplification
P240	P240_1	67.5	Neuroendocrine	Bone, lymph nodes, liver, lung	Yes	NA	3.2	542.4	Carboplatin/Etoposid	ADT, Chemotherapy	No
P198	P198_5	65.1	Neuroendocrine	Bone, lymph nodes	Yes	NA	29.4	> 370	ADT	ADT, Chemotherapy, Carboplatin/Etoposid	No
P190	P190_3	65.4	Adenocarcinoma/ Neoroendocrine	Bone	Yes	NA	3.35	19.4	Carboplatin/Etoposid	ADT, Chemotherapy	No
P179	P179_4	64.9	Neuroendocrine	Bone, lymph nodes	Yes	15.0	0.56	55.6*	Chemotherapy	ADT	No
P170	P170_2	72.4	Neuroendocrine	Bone, lung, liver	Yes	NA	3.81	122.6	Carboplatin/Etoposid	ADT, Chemotherapy	No
P148	P148_3	66.0	Neuroendocrine		Yes	NA	52.0	> 370	Carboplatin/Etoposid	Chemotherapy	No
	P148_1		Adenocarcinoma	Bone, liver, lymph nodes	Yes		694.4	NA	Chemotherapy	ADT	Yes
P147	P147_3	65.5	Adenocarcinoma	Bone, liver, lymph nodes	Yes	11.4	863.1	> 370	Chemotherapy	ADT, RT	Yes
	P147_1				Yes		205.7	NA			Yes
P40	P40_2	84.1	Adenocarcinoma	Bone	Yes	425.3	656.0	NA	ADT	RT	Yes
	P40_1				No		115.3	NA			No

Breast cancer patients

Patient ID	Sample ID	Age (years)	Tumor type	Her2-	ER+ Intermediate	PR+low	PR+ Intermediate
B7	B7_1	54.4861111111111	luminal	1	1	1	
B13	B13_1	75.4305555555556	luminal	1	1		1

Colon cancer patient

Patient ID	Sample ID	Age (years)	Tumor type	Metastatic sites	UICC
C2	C2_7	48	Adenocarcinoma	Liver	G3 pT3 N2b V1
	C2_6				

Clinical data on prostate cancer patients P40, P147, P148, P190, P170, P179, P198, and P240, on breast cancer patients B7 and B13, and patient C2 with colorectal cancer.

Supplementary Table 1

Supplementary Table 2

	Control samples	Cancer samples
**n**	177	592
**male**	36.36%	51.78%
**female**	63.64%	48.22%
**stage I**	n/a	197
**stage II**	n/a	280
**stage III**	n/a	98
**stage IV**	n/a	6
**stage NA**	n/a	9
**Mean (+/- sd)**	53.1 (+/- 15.98)	70.6 (+/- 11.50)

Clinical characteristics of the Freenome colorectal cancer cohort.

Supplementary Table 3

	StageI		StageII		StageI+II		All Samples	
	Value	95%-Conf Int	Value	95%-Conf Int	Value	95%-Conf Int	Value	95%-Conf Int
**Samples in Cancer set**	197		280		477		592	
**Accuracy**	0.718	0.565-0.842	0.755	0.619-0.881	0.736	0.636-0.841	0.728	0.630-0.818
**Precision**	0.742	0.558-0.905	0.838	0.689-1.0	0.859	0.764-0.966	0.861	0.748-0.946
**Recall**	0.711	0.538-0.873	0.745	0.575-1.0	0.766	0.649-0.861	0.771	0.664-0.870
**Specificity**	0.724	0.480-0.898	0.771	0.563-1.0	0.658	0.427-0.894	0.59	0.304-0.823
**F1**	0.721	0.558-0.845	0.785	0.652-0.898	0.808	0.726-0.893	0.812	0.738-0.883
**MCC**	0.436	0.123-0.690	0.506	0.216-0.761	0.393	0.143-0.643	0.327	0.048-0.520
**AUC**	0.793	0.664-0.902	0.827	0.684-0.931	0.775	0.636-0.895	0.762	0.602-0.867

Performance metrics of logistic regression analysis of TF accessibilities in the Freenome cohort of early-stage colorectal cancer samples.
