# Supplementary material for: Inference of transcription factor binding from cell-free DNA enables tumor subtype prediction and early detection
Source: Nat Commun. 2019 Oct 11;10:4666. doi: 10.1038/s41467-019-12714-4 (PMC6789008; doi:10.1038/s41467-019-12714-4)
Supplement: Supplementary file 3 — Description of Additional Supplementary Files [file 41467_2019_12714_MOESM3_ESM.pdf]

Description of Additional Supplementary Files

File name: Supplementary Data 1
Description: List of 504 TFs used in this study and accompanying information.

File name: Supplementary Data 2
Description: Ranking order of TF accessibilities for prostate, colon, and breast epithelial tissues established from ATAC-seq data.

File name: Supplementary Data 3
Description: Overview of samples and sequencing statistics with accompanying information about tumor fractions from ichorCNA.

File name: Supplementary Data 4
Description: Overall z-scores for individual samples, merged low-coverage samples and downsampled samples of P148.
